# Time to Service and Its Relationship with Outcomes in Workers with Compensated Musculoskeletal Conditions: A Scoping Review

**DOI:** 10.1007/s10926-023-10160-0

**Published:** 2024-01-12

**Authors:** Tesfaye Hambisa Mekonnen, Michael Di Donato, Alex Collie, Grant Russell

**Affiliations:** 1 Department of General Practice, School of Public Health and Preventive Medicine, Monash University, 553 St Kilda Road, Melbourne, 3004 Australia; 2 Healthy Working Lives Research Group, School of Public Health and Preventive Medicine, Monash University, 553 St Kilda Road, Melbourne, 3004 Australia

**Keywords:** Time-to-service, Musculoskeletal condition, Low back pain, Workers' compensation

## Abstract

**Purpose:**

A comprehensive review of the literature on the time between the onset of symptoms and the first episode of care and its effects on important worker outcomes in compensated musculoskeletal conditions is currently lacking. This scoping review aimed to summarize the factors associated with time to service and describe outcomes in workers with workers’ compensation accepted claims for musculoskeletal conditions.

**Methods:**

We used the JBI guidelines for scoping reviews and reported following the PRISMA-ScR protocol. We included peer-reviewed articles published in English that measured the timing of health service initiation. We conducted searches in six databases, including Medline (Ovid), Embase (Ovid), PsycINFO, Cinahl Plus (EBSCOhost), Scopus, and the Web of Science. Peer-reviewed articles published up to November 01, 2022 were included. The evidence was summarized using a narrative synthesis.

**Results:**

Out of the 3502 studies identified, 31 were included. Eight studies reported the factors associated with time to service. Male workers, availability of return to work programmes, physically demanding occupations, and greater injury severity were associated with a shorter time to service, whereas female workers, a high number of employees in the workplace, and having legal representation were associated with a longer time to service. The relationship between time service and worker outcomes was observed in 25 studies, with early access to physical therapy and biopsychosocial interventions indicating favourable outcomes. Conversely, early opioids, and MRI in the absence of severe underlying conditions were associated with a longer duration of disability, higher claim costs, and increased healthcare utilization.

**Conclusion:**

Existing evidence suggests that the time to service for individuals with compensated musculoskeletal conditions was found to be associated with several characteristics. The relationship between time to service and worker outcomes was consistently indicated in the majority of the studies. This review highlights the need to consider patient-centred treatments and develop strategies to decrease early services with negative effects and increase access to early services with better outcomes.

**Supplementary Information:**

The online version contains supplementary material available at 10.1007/s10926-023-10160-0.

## Background

Work-related musculoskeletal conditions are a major cause of work disability worldwide [1]. These conditions constitute a considerable proportion of workers’ compensation claims [2–4]. In Canada and Australia, an estimated 1.2 million claims involving time off work were compensated for musculoskeletal conditions between 2004 and 2013 [5]. In Australia, injury and musculoskeletal conditions contributed to 87% of all workers’ compensation claims in 2020–2021 [6]. Despite a decreasing trend in total claim rates, work disability (i.e. absence from work due to injury/illness) and related compensation costs for musculoskeletal conditions remain a significant problem in high-income countries. For example, total workers’ compensation costs in Australia have increased 30% since 2016–2017 to $10.8 billion in 2020–2021 [4, 7]. In 2021–2022, musculoskeletal conditions represented approximately 7.3 million cases of time loss from work in Great Britain [8]. Most workers’ compensation schemes fund healthcare services to support injured workers’ return to work and recovery trajectories [9–11].

The timing of healthcare service is a key quality indicator for process measures within workers’ compensation systems [12] and has previously been associated with outcomes for workers with claims for musculoskeletal conditions [13, 14]. For example, several studies have demonstrated that delay in appropriate health services is associated with a longer period of absence from work (i.e. extended duration of disability), poorer rates of return to work, and worse recovery outcomes for musculoskeletal conditions, such as low back pain [15–19]. A recent cohort study on the timing of physical therapy among individuals with knee osteoarthritis demonstrates that a delay in initiating physical therapy of more than one month is associated with an increased risk of future opioid utilization compared to initiating physical therapy within one month (i.e. early) of the index date [20].

Timely access to appropriate healthcare services can expedite injury recovery and facilitate a quicker return to work [21–23]. Findings from a randomized controlled trial study reveal that early intervention, involving thorough examinations, information, and recommendations to stay active for patients with acute low back conditions, resulted in a significantly higher return to work rate at 12-month follow-up (i.e. 68.4% of the patients in the intervention group returned to work compared to 56.4% in the control group) [24]. Moreover, a systematic review of physical therapy (PT) studies by Ojha and Colleagues found that early PT, compared to delayed PT, was associated with lower costs and reduced subsequent health service utilization [25]. However, it is important to note that early treatment with some services with limited evidence to support, such as opioids and magnetic resonance imaging (MRI) for some musculoskeletal conditions (e.g. acute nonspecific low back condition), is not always useful and can result in increased healthcare costs and utilization [26]. A recent systematic review and narrative analysis showed that undergoing early MRI (i.e. MRI within the first 4 to 6 weeks of the index visit) compared to no MRI for low back pain without severe underlying conditions is associated with a longer disability duration [27]. Moreover, another systematic review discovered that prescribing opioids within the first 12 weeks (early) of the onset of musculoskeletal conditions is associated with prolonged work disability among workers’ compensation claims [28].

Access to timely and appropriate healthcare services within the workers’ compensation system can be influenced by various factors. Individual characteristics (e.g. age and gender), injury severity, occupation, and provider type have been previously reported as the factors that can affect the timing of health service utilization [29]. Furthermore, factors related to insurance policies (e.g. waiting periods for assessment, financial incentives, limiting provider choice in some jurisdictions), healthcare-related factors (e.g. health providers’ unwillingness to treat patients receiving workers’ compensation), work-related factors (e.g. work-relatedness of the injury), and access challenges (e.g. remoteness) have been shown to influence the time to service [15, 18, 22, 29–32]. A study conducted by Kominski and Colleagues revealed that policies that limit healthcare utilization may have a negative impact on access to quality care, return to work rates, and recovery outcomes [15]. In the United States, for example, due to limited first-line provider choice, 13.3% of workers encountered “some or a lot of difficulty getting medical care” when they were first injured [33]. Similarly, a study in California found that 8.5% of workers faced challenges in accessing physical therapists, 7.9% “specialist care”, or 2.5% “prescription medications” [15]. While several reviews have been conducted in the general population [34, 35], there is a lack of evidence regarding a comprehensive review of health service timing and the factors influencing the timing of health services for musculoskeletal conditions within the context of workers’ compensation systems exclusively.

Given the pervasive nature of musculoskeletal conditions and the corresponding WC claims, it is paramount to systematically map the available literature regarding the factors that influence compensation outcomes and the relationship between time-to-service and those outcomes. Empirical data on the timing of health service differ in terms of musculoskeletal conditions, types of services, and the outcome measures involved, and aggregating findings of multiple studies is impractical [27, 28, 36]. As a result, we conducted a scoping review to provide a literature summary of the factors influencing time-to-service and describe the time-to-service relationship with worker outcomes. To inform better healthcare funding practices, a comprehensive overview of the literature regarding the factors influencing time to service and its relationship with outcomes among workers with compensated musculoskeletal conditions is needed.

**Research questions**

i. What factors are associated with time to service in studies of individuals with musculoskeletal conditions and accepted workers’ compensation claims?
ii. What is the association between time to service and work and health outcomes in those individuals?

## Methods

This scoping review study followed the Joanna Briggs Institute (JBI) framework [37] and was reported using the Preferred Reporting Items for Systematic Reviews and Meta-analysis Extension for Scoping Reviews (PRISMA-ScR) (Supplementary file 1) [38]. The review protocol was pre-registered with the Open Science Foundation (link: https://osf.io/xjyd8).

### Eligibility Criteria

#### Participants

Workers aged 15 years and above with an accepted workers’ compensation claim for a musculoskeletal condition affecting any body region were included [39]. Work-related musculoskeletal conditions at any stage of progression (i.e. acute, subacute, or chronic) were examined for inclusion.

#### Concept

We included studies that reported the time between an initial event, such as the initial report of musculoskeletal complaints, the date of claim acceptance, or primary index date and the services provided (time to service). We reviewed studies involving any treatment service (e.g. pharmacological and nonpharmacological) and diagnostic service (e.g. magnetic resonance imaging (MRI), X-ray, and ultrasound) funded by workers’ compensation. Evidence where the duration/average duration between the onset of injury and the first episode of care was not specified, and contact with healthcare providers for purposes other than treatment/diagnostic services (e.g. injury report writing and independent medical evaluations) were excluded.

#### Context

Personal injury reports involving transportation accidents (motor vehicle), the military, sports, and daily/home activities were excluded because injury cases in these settings are typically handled through alternative compensation schemes.

### Type of Evidence Sources

Peer-reviewed studies published in English, including randomized and non-randomized controlled trials, prospective and retrospective cohorts, case-control, analytical cross-sectional, and qualitative studies, were considered. Expert comments, perspective papers, conference abstracts, editorials, supplements, and magazine reports were excluded. Grey literature, such as dissertations and national survey data, was also excluded. Citation chaining was used to identify missing pertinent articles [40, 41].

### Changes from the Original Protocol

Minor changes were made to the inclusion criteria indicated in the registered protocol. First, our preliminary search indicated that the working age group in certain important industries was older than 65, which was our original maximum cut-off age limit. As a result, we did not limit the maximum age in the review to 65 years. Second, grey literature, such as dissertations and national survey reports, expert opinions, viewpoint papers, and conference abstracts, was excluded from this review as we identified sufficient peer-reviewed literature to address our research questions. Third, the most recent search date for all databases included in this review (November 1, 2022) occurred after the date specified in the protocol (September 1, 2022). Finally, we added certain items to the protocol’s data charting table as new findings became available.

### Search Strategy

**Fig. 1:**
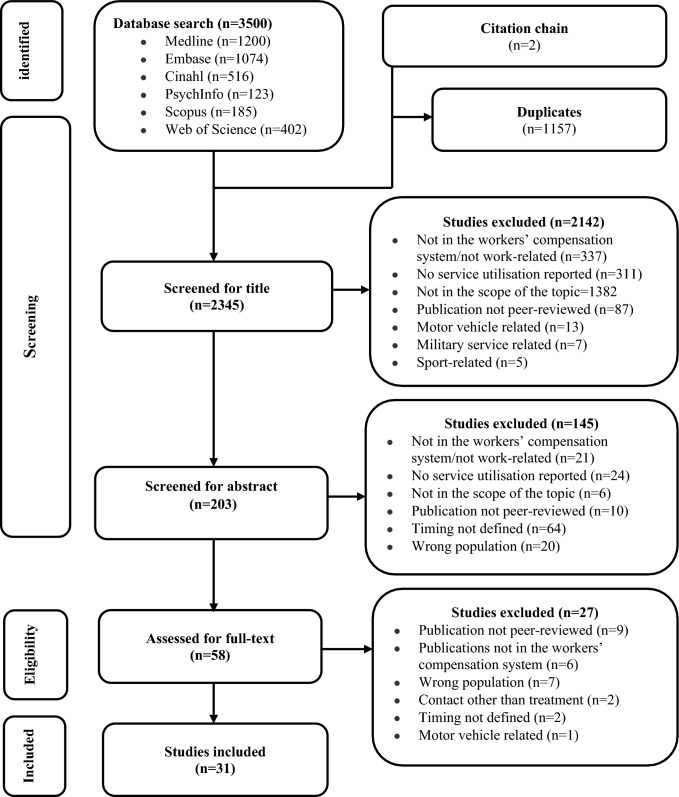
PRISMA flow chart (n = 31)

A preliminary search was conducted in the Medline (Ovid) database to identify text words and index keywords using the participant, concept, and context (PCC) approach [42]. Synonyms of musculoskeletal injury, time-to-treatment, work-related injury, and workers’ compensation were used. Terms of related concepts were combined using the Boolean OR operator, whereas the Boolean AND operator was used to combine different concepts. The search strategy was developed by two authors (THM and MDD) and was reviewed by a third author (GR) in consultation with a professional librarian. The final search was conducted on November 1, 2022, in six databases: MEDLINE (Ovid), EMBASE (Ovid), Psych Info (Ovid), Cumulative Index to Nursing and Allied Health Literature (CINAHL), Scopus, and Web of Science. Peer-reviewed studies published in English until November 01, 2022, were included. The PRISMA flow diagram **(**Fig. 1**)** displays the evidence screening steps, and Supplementary File 2 provides the Medline search strategy.

### Study Selection

Citation management was conducted using Endnote software version 19.3 [43]. The citations were then exported to Covidence® for duplicate removal and evidence screening [44]. Two reviewers (THM and MDD) screened the titles and abstracts independently. The full-text studies that passed the initial screening step were obtained, and the same reviewers independently screened the full-text articles. Articles that did not fit the inclusion criteria were removed, and the reason was documented. Minimal disagreements were settled through discussion at all steps. Three authors (THM, MDD, and GR) were involved in the decision-making process regarding article exclusions.

### Data Extraction/Charting

Data charting was conducted in Microsoft Excel using a standard data extraction template (Supplementary file 3). After checking for the comprehensiveness of the pilot findings conducted by one reviewer (MDD), another reviewer (THM) completed the entire data extraction. One reviewer (MDD) double-checked the extraction of seven articles at random, and the result was consistent between the authors. The characteristics of the study, including first author, year, title, journal, country, study design, data source, inclusion and exclusion criteria, study sample and sample size, and participant characteristics such as sex, age (mean with standard deviation, median with interquartile range), type of musculoskeletal conditions, type of services, main findings, and author conclusions were extracted. We charted detailed data regarding the use of time-to-service in each study (i.e. as an outcome, predictor, or both), information on the type of timing measure (e.g. continuous, categorical relative to a goal or certain treatment guideline, such as ‘early’), timing measurement (e.g. hours, days, weeks, and months ), timing start point, time-to-service average duration, factors affecting time-to-service and findings, and time-to-service predicting outcomes.

### Summarizing and Reporting the Results

We first described the characteristics of the included studies. We then developed a narrative summary of time to service, followed by a synthesis table with the average duration and timing definitions. Next, we conducted a narrative summary of factors affecting time-to-service. Factors affecting time-to-service were categorized into four themes: individual, injury-related, workplace, and health services-related factors. The selection of the variables is based on the Behavioural Model of Health Services utilization, with a slight modification made to accommodate variables available within the workers’ compensation system administrative data, including work-related factors [45]. A descriptive summary of study outcomes (e.g. disability duration) was also produced, and the relationship between time to service and outcomes was described. We grouped worker outcomes into four categories following a previous study approach [25]: work outcomes, claim costs, healthcare utilization, and patient-reported health outcomes. Additionally, a summary table that includes the relationship between the outcomes and time to service, definitions of outcomes, and some study features was developed. At each stage of the process, the results were reviewed, refined, and feedback was shared among the authors until a final agreement was reached.

## Results

### Studies Selection

Electronic database searches yielded 3500 references in total. Citation chaining returned two additional relevant articles. After removing duplicates, 2345 records progressed to the title and abstract screening. Following title and abstract screening, 58 reports were passed for full-text review. During the full-text screening, 27 citations were excluded, leaving 31 eligible articles for inclusion. The PRISMA flow diagram **(**Fig. 1**)** fully reports the search results [46].

### Study Characteristics

Table 1 presents the key characteristics of the included studies. Studies originated from the United States (*n* = 22) [40, 41, 47–66] and Canada (*n* = 9) [67–75], with most published from 2000 onwards (*n* = 27) [40, 41, 47–50, 52, 54, 55, 57–67, 69, 71–73, 75–77]. In (*n* = 5) studies, the study inception period/year of injury occurred before 2000 [53, 56, 64, 68, 74], and no year of injury was specified in (n = 2) studies [65, 67]. A retrospective cohort was the most common study design reported (n = 22) [40, 41, 47, 48, 54, 56–65, 69–75]. Other included studies used a prospective cohort (*n* = 6) [50–52, 55, 66, 68], randomized controlled trial (RCT) (*n* = 2) [53, 67], and cross-sectional (*n* = 1) [49] methods. Most studies used administrative data directly from workers’ compensation schemes (*n* = 20) ) [40, 41, 47, 48, 51, 54, 56–65, 69, 71, 75, 76] or with other studies using data sources such as employee interviews, medical records, and surveys (*n* = 11) [49, 50, 53, 55, 66–68, 72–74, 78]. The sample size ranged from 63 [67] to 137,175 participants [75].

**Table 1:** Characteristics of the included studies (n*=31)

Author (country)	Year of injury	Design	Data source	Sample size (*n*)	Sex (%)	Age (mean + SD) in years	Type of musculoskeletal conditions	Type of services	Use of time to service^@^
Besen et al. 2016 (USA) [47]	January 1, 2002 to December 31, 2008	Retrospective cohort	Workers’ compensation (WC) administrative data	64,004	Male (69.4)	40 (11.2)	Low back pain (based on the international classification of diseases (ICD-9 codes)	Any visit	Predictor
Besen et al. 2018 (USA) [48]	January 1, 2002 to December 31, 2008	Retrospective cohort	Workers’ compensation (WC) administrative data	76,067	Male (66.3)	42.4(11.9)	Work-related musculoskeletal diseases and fractures international classification of diseases (ICD-9 diagnosis codes)	Any visit	Predictor
Blanchette et al. 2017 (Canada) [70]	January 01, 2005 to June 30, 2005	Retrospective cohort	Workers’ compensation (WC) administrative data	5,520	Male (61.9)	NR	Uncomplicated back pain	Multiple*	Both
Busse et al. 2015 (Canada) [71]	January 01, 2005 to June 30, 2005	Observational cohort study	WC administrative data	1442	Male (61.7)	41.3(10.5)	Low back pain	Multiple	Predictor
Carnide et al. 2020 (Canada) [72]	1998 to 2009	Historical cohort	WC administrative data and healthcare data	54,130	Male (63.1)	41.1(10.9)	Low back pain	Opioid	Outcome
Carnide et al. 2019 (Canada) [73]	1998 to 2009	Historical cohort	WC administrative data linked with Population	55,571	Male (63.1)	NR	Low back pain	Opioids	Predictor
Cote et al. 2005 (USA) [49]	July 01, 1999 to June 30, 2002	Cross sectional	WC administrative data and interview	1104	NR	NR	Back pain (with or without leg pain or sciatica)	Multiple services	Outcome
Ehrmann-Feldman et al. 1996 (Canada) [74]	1988	Retrospective cohort	Claim data, medical files, and initial reports completed by physical therapist	2147	Female (23)	36.4	Back pain	Physical therapy	Predictor
Faour et al. 2017 (USA) [41]	1993 to 2011	Retrospective cohort	WC administrative data	1509	Radiculopathy: female (33.2) DDD: female (38.2)	Radiculopathy: 44(8) DDD:46(8)	Radiculopathy (Neck Pain with Radicular Symptoms) and Degenerative Disc Disease (discogenic neck pain)	Surgery	Predictor
Franklin et al. 2008 (USA) [50]	July 2002 to April 2004	Prospective cohort	WC administrative data and interview	1843	Male (68)	39.4(11.2)	Low back pain	Opioids	Predictor
Graves et al. 2012 (USA) [77]	July 2002 to April 2004	Prospective cohort	WC administrative data	1830	Female (32)	39.4(11.2)	Low back pain	MRI	Both
Graves et al. 2014 (USA) [52]	July 2002 to April 2004	Prospective cohort	WC administrative data and interview	1770	Female (27.1)	NR	Low back pain	MRI	Predictor
Greenwood et al. 1990 (USA) [53]	1985 to 1986	Controlled study	WC administrative data and interview	284	Female (3)	39	Low back pain	Biopsychosocial (case management supported)	Predictor
Gross et al. 2009 (Canada) [75]	January 01, 2000 December 31, 2005	Historical cohort study	WC administrative data	137,175	Male (70)	37	Back and other sprain/strain, fracture, amputation, burn, or dislocation	Opioid	Both
Haight et al. 2020 (USA) [62]	July 01, 2002 to August 30, 2013	Retrospective cohort	WC administrative data	83,150	Male (69)	36.9	Lower extremity, upper extremity, back/neck, other/multiple); acute/sprain/strain/tears, fractures, traumatic injuries,	Opioid	Predictor
Heins et al. 2016 (USA) [61]	June 01, 1999 to June 01, 2010	Retrospective cohort	WC administrative data	123,096	NR	NR	Back and shoulder pain	Opioid	Predictor
Lavin et al. 2013 (USA) [63]	1999 to 2002	Retrospective cohort	WC administrative data	582	NR	NR	Low back pain	Surgery	Predictor
Mahmud et al. 2000 (USA) [64]	June 01, 1995 to August 31, 1995	Retrospective cohort	WC administrative data	98	Male (71.4)	37	Low back pain	MRI	Predictor
Patel et al. 2022 (USA) [65]	Not reported	Retrospective cohort	WC administrative data	193	NR	NR	Low back pain (degenerative spinal disease)	Lumbar surgery	Predictor
Phillips et al. 2017 (USA) [66]	January 2012 to June 2013	Prospective pilot study	WC administrative data and patients’ chart	75	Pilot cohorts: Female (80) Nonpilot cohorts: NR	Pilot cohorts: 40.4 (11.6) years Nonpilot cohorts: 43.2 (14.4) years	Musculoskeletal injuries of sprain, strain, pain, contusion, numbness, cumulative trauma, tendonitis, muscle spasm, and inflammation (ICD-9 codes)	Physical therapy	Both
Razmjou et al. 2015 (Canada) [69]	November 2012 to July 2014	Retrospective cohort	WC administrative data	550	Male (53)	49(11)	Shoulder pain	Multiple	Predictor
Ren et al. 2020 (USA) [40]	1993 to 2013	Retrospective cohort	WC administrative data	791	NR	NR	Low Back Pain	Surgery	Both
Schultz et al. 2013 (Canada) [67]	Not reported	Randomized controlled trial	WC administrative data and interview	63	NR	NR	Low Back Pain	Interdisciplinary biopsychosocial approach	Predictor
Sinclair et al. 1997 (Canada) [68]	May to November 1993	Prospective cohort	WC administrative data and interview	885	NR	NR	Soft tissue musculoskeletal conditions, including the back, upper or lower limb	Physical therapy	Predictor
Sinnott et al. 2009 (USA) [54]	January 01, 1993 to December 31, 2000	Retrospective cohort	WC administrative data	35,304	Female (25.4)	36.87(10.95)	Low back pain	Any visit	Predictor
Stover et al. 2006 (USA) [55]	July 01, 2002 to June 30, 2003	Prospective cohort	Medical records, administrative data, and interviews	1067	Male (69.3)	NR	Low back pain	Opioids	Outcome
Tacci et al. 1998 (USA) [56]	June 01, 1995 to August 31, 1995	Retrospective cohort	WC administrative data	98	Male (72.5)	34(11)	Low back pain	Multiple	Predictor
Wasiak et al. 2007 (USA) [57]	January 1, 1997 to June 30, 2001	Retrospective cohort	WC administrative data	6019	Female (29.3)	38.2(11.8)	Low Back Pain	Chiropractor	Predictor
Webster et al. 2010 (USA) [58]	January 01, 2006 to December 31, 2006	Retrospective cohort	WC administrative data	8443	Male (69.7)	41.4	Low Back Pain	MRI	Predictor
Webster et al. 2014 (USA [68]	January 01, 2006 to December 31, 2006	Retrospective cohort	WC administrative data	3022	NR	NR	Low Back Pain	MRI	Predictor
Webster et al. 2007 (USA) [59]	January 01, 2002 to December 31,2003.	Retrospective cohort	WC administrative data	3264	Female (28.2)	40.3 (10.4)	Low Back Pain	Opioid	Predictor

* use of time to service either as a predictor, outcome, or both DDD Degenerative Disk Disease, ICD International classification of Diseases, n* number of studies, n Sample size, NR not reported, USA United states of America, WC Workers’ compensation

### Characteristics of Musculoskeletal Condition

**Fig. 2:**
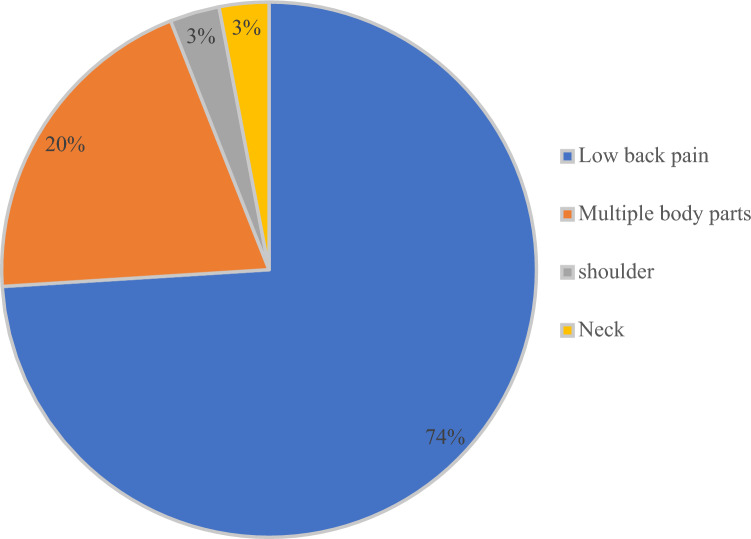
Musculoskeletal conditions included (*n* = 31)

A large number of studies included workers with low back pain in (*n* = 23) studies [40, 47, 49–60, 63–65, 67, 70–74]. More than one condition (multiple body parts) was reported in (*n* = 6) studies [48, 61, 62, 66, 68, 75], and (n = 1) study each for shoulder pain [69] and neck pain [41](Fig. 2).

### Description of Time to Service

Time to service was most commonly used as a predictor in (*n* = 23) studies [41, 47, 48, 50, 52–54, 56–65, 67–69, 71, 73, 74] in reporting rather than as an outcome in (*n* = 3) studies [49, 55, 72]. Time to service was used as both a predictor and an outcome in (*n *= 5) studies [40, 51, 66, 70, 75]. Duration of time to service was measured from the date of injury in (*n* = 25) studies [40, 41, 47–49, 51–54, 56, 58–65, 67–70, 72–74], claim acceptance date in (*n* = 2) studies [71, 75], and index visit (i.e. first service) was used in (*n* = 4) studies [50, 55, 57, 66].

Measures of time to services varied depending on the type of services and musculoskeletal conditions involved. Several studies reported service timing categorically (or in binary terms) by classifying a service as either early or not. This usually occurred in studies of opioid, MRI, and physical therapy services for low back pain, where the measure of whether a service was early was based on guideline recommendations or evidence. For example, five studies involving magnetic resonance imaging (MRI) defined early service as service received within six weeks (*n* = 3) [52, 64, 77] and within the first 30 days (*n* = 2) [58, 60] of back conditions.

Overall, there was no standard timing definition, even for a particular service and condition. In addition, the reason for choosing different timing classifications within cohorts has not been described in some studies (Table 2**)**.

**Table 2:** Duration and definitions of health service timing (n*=31)

Author (year) and Region	Inclusion/participants	Type of musculoskeletal conditions	Service	The average duration of service initiation	Timing definition
Besen et al. 2016 [47] (USA)	Workers 18 to 80 years of age, at least one day of paid lost work time within one year of the injury date, and had only one claim within a single calendar year, multiple episodes of disability within the same claim had less than 14 days between episodes	Low back pain (based on the international classification of diseases (ICD-9 codes)	Any visit	0 days medical service lag, 1 to 3 days lag, 4 to 6 days lag, 1 week up to 2 weeks lag, 2 weeks up to 30 days lag, 30 days up to 60 days lag, and 60 days up to 1-year lag.	Medical service lag time (Shorter lag time (0-day lag time (reference) versus longer lag time 60 days up to 1-year lag time to medical services)
Besen et al. 2018 [48] (USA)	Workers with at least one day of paid lost work time, ages 18 to 80 at the time of injury, had only one claim and only one episode of disability for a single claim within a calendar year	Work-related musculoskeletal diseases and fractures international classification of diseases (ICD-9 diagnosis codes)	Any visit	0 to 1-day lag, 2 to 6 days lag, 1 week up to 2 weeks lag, 2 weeks up to 30 days lag, and 30 days up to 1-year medical service lag time	Shorter versus longer treatment lag time based on a priori defined group of periods
Blanchette et al. 2017 [70] (Canada)	Workers with a lost-time claim	Uncomplicated back pain	Multiple	Service within a month after injury	Definition not reported
Busse et al. 2015 [71] (Canada)	Workers who remained on full benefits at four weeks after claim approval	Low back pain	Multiple	Reimbursement within four weeks of claim approval	Early
Carnide et al. 2020 [72] (Canada)	Workers aged ≥ 18, one day income replacement benefit in the first eight weeks of onset, no back-related hospitalization or serious back-related outpatient service within five days after injury, and resident of British Columbia two years before and one year after injury	Low back pain	Opioid	The first eight weeks after injury	Early
Carnide et al. 2019 [73] (Canada)	Accepted claims for LBP, had at least one day of wage replacement benefits were not consolidated claims (e.g. duplicate claims) and had no low back-related hospitalization and/or serious outpatient service within five days after injury, age 18 years and older, and British Columbia residents two years before through one year after injury, injury date equal or precede claim registration date	Low back pain	Opioids	Prescription within eight weeks after the injury date	Early
Cote et al. 2005 [49] (USA)	Age ≥ 18, made claim for work-related back pain (with or without leg pain or sciatica) between July 1, 1999 and June 30, 2002	Back pain (with or without leg pain or sciatica)	Multiple services	Service (genera) within 16 weeks of injury date	Early
Ehrmann-Feldman et al. 1996 [74] (Canada)	Workers with no compensation for a back injury in the two years (1986 and 1987) before the study, at least one full day of absence from work, and ages of 15 and 65 years	Back pain	Physical therapy	Within 1 month of the injury date	Early
Faour et al. 2017 [41] (USA)	Patients who underwent multilevel cervical fusion for Radiculopathy (Neck Pain with Radicular Symptoms) and Degenerative Disc Disease (discogenic neck pain) and had at least 3 years of follow-up after Surgery	Radiculopathy (Neck Pain with Radicular Symptoms) and Degenerative Disc Disease (discogenic neck pain)	Surgery	Services within greater than two years of injury date	Definition not reported
Franklin et al. 2008 [50] (USA)	Workers who had an accepted claim, had four or more days of lost time from work, at least 18 years of age, enrolled in the study and completed a baseline telephone interview; received at least one day of wage replacement compensation in the first year of the claim, and were not hospitalized in the acute period after the injury	Low back pain	Opioids	Opioids within six weeks of a first medical visit	Early
Graves et al. 2012 [77] (USA)	Age ≥ 18, accepted claim, received compensation for missing four or more days from work, and not hospitalized in the acute period after low back injury	Low back pain	MRI	MRI (yes/no), defined as receiving a lumbar MRI 42 or fewer days/ within 6 weeks after injury date	Early
Graves et al. 2014 [52] (USA)	Workers who were 18 years and older and greater and more than four days of lost work time	Low back pain	MRI	MRI within six weeks of the injury date	early (nonadherent MRI)
Greenwood et al. 1990 [53] (USA)	Compensated workers with a new claim for back injury	Low back pain	Biopsychosocial (case management supported)	Service within two weeks after injury date	Early
Gross et al. 2009 [75] (Canada)	Workers with time loss claim for back and other sprain/strain, fracture, amputation, burn, or dislocation	Back and other sprain/strain, fracture, amputation, burn, or dislocation	Opioid	Prescription within first two weeks of claim acceptance	Early
Haight et al. 2020 [75] (USA)	Workers with accepted claims,18–56 years of age, had at least one opioids prescription within the first six weeks of the date of injury, the first attending primary care provider (authorized to prescribe opioids)	Lower extremity, upper extremity, back/neck, other/multiple); acute/sprain/strain/tears, fractures, traumatic injuries,	Opioid	Prescription within 6 weeks of injury date	Early
Heins et al. 2016 [61] (USA)	Compensated who received medical and indemnity payments	Back and shoulder pain	Opioid	Opioids prescription dispensed within ≤ 1 month of injury date	Early
Lavin et al. 2013 [63] (USA)	A cohort of all lost time involving lumbar spinal surgery	Low back pain	Surgery	Service within 1 to 89 days, 90 to 179 days, 180 to 359 days, and 360 + days	Early versus delay timing defined and compared within group
Mahmud et al. 2000 [64] (USA)	Workers with acute, uncomplicated disabling low back pain with lost time work	Low back pain	MRI	Within 30 days of injury date	Early
Patel et al. 2022 [65] (USA)	Claimants who underwent minimally invasive transforaminal lumbar interbody fusion	Low back pain (degenerative spinal disease)	Lumbar surgery	Timing categorized in to: < 90 days, 90–179 days, and > 180 days of the first appointment and within < 180 days, 180–364 days, and ≥ 365 days of the first symptom reported	Longer versus shorter
Phillips et al. 2017 [66] (USA)	Compensated workers who had musculoskeletal injuries of sprain, strain, pain, contusion, numbness, cumulative trauma, tendonitis, muscle spasm, and inflammation (ICD-9 codes)	Musculoskeletal injuries of sprain, strain, pain, contusion, numbness, cumulative trauma, tendonitis, muscle spasm, and inflammation (ICD-9 codes)	Physical therapy	Physical therapy service utilized at the point of initial injury care	Early
Razmjou et al. 2015 [69] (Canada)	Workers with a shoulder injury and had an accepted claim	Shoulder pain	Multidisciplinary	≤ 16 and > 16 weeks of the injury date	Early (service used within ≤ 16 weeks of injury onset)
Ren et al. 2020 [40] (USA)	Workers with low back (spondylolisthesis) who received a lumbar fusion between 1993 and 2013	Low Back Pain	Surgery	Service within two years and after two years of injury	Shorter (≤ 2 years) versus longer (> 2 years)
Schultz et al. 2013 [67] (Canada)	Off work for four to ten weeks post low back pain injury and at high risk and moderate risk of disability, ages 19–65, have worked less than 20 h/week, were in receipt of workers’ compensation temporary partial or total disability benefits, and were able to read and respond in English	Low Back Pain	Interdisciplinary biopsychosocial approach	Within four to ten weeks after injury date	Early
Sinclair et al. 1997 [68] (Canada)	An accepted new claim for soft tissue musculoskeletal conditions, including the back, upper or lower limb	Soft tissue musculoskeletal conditions, including the back, upper or lower limb	Physical therapy	Early as two days and as delay as seventy days of injury date	Early
Sinnott et al. 2009 [54](USA)	Workers who received indemnity benefits for more than three days of temporary disability for acute low back pain	Low back pain	Any visit	≤ 14 days; 14 and ≤ 28 days; 28 and ≤ 56 days; > 56 and ≤ 84; > 84 and ≤ 182 days; and > 82 days	Delays defined compared against within the group where delay ≤ 14 days was a reference
Stover et al. 2006 [55] (USA)	Compensated workers with acute low back pain, 18 years or older, and had at least four days of work disability	Low back pain	Opioids	Prescription within six weeks of first medical visit	Early
Tacci et al.1998 [56] (USA)	Workers with lost time acute, uncomplicated low back pain claims that had associated medical costs claims initiated between June 1, 1995, and August 31,1995	Low back pain	Multiple	The first contact (any visit) started with a mean of 2.8 days and a median of 1 injury. first physician visit made within a mean of 3.5 days and median of 1 day of injury	The early pattern of healthcare use, including the referral system
Wasiak et al. 2007 [57] (USA)	Workers with low back pain who had at least one visit to a chiropractor during a 4-year period after the reported date of first symptom onset	Low Back Pain	Chiropractor	Service is received within 30 days of the beginning of the episode of care and more than 30 days from the beginning of the episode of care.	Early versus late
Webster et al. 2010 [58] (USA)	Compensated workers with low back pain, one day or more of compensated lost time within the first 10 days post-onset and at least one year of job tenure	Low Back Pain	MRI	MRI Service within 30 days of injury date	Early
Webster et al. 2014 [60] (USA)	Workers with at least one day compensated lost time and at least one year of job tenure	Low Back Pain	MRI	MRI received within 30 days of injury date)	Early
Webster et al. 2007 [59] (USA)	Workers with one day or more of compensated lost time for new onset, disabling back who had no back claims in the prior year, whose lost time began within 10 days of low back pain onset, and who received at least one paid medical service within 15 days post-onset (occurred between January 1, 2002, and December 31, 2003)	Low Back Pain	Opioid	Within 15 days of injury	Early

ICD International classification of Diseases, n* number of studies, RCT Randomized controlled trial, USA United states of America, WC Workers’ compensation

### Description of the Included Services

**Fig. 3:**
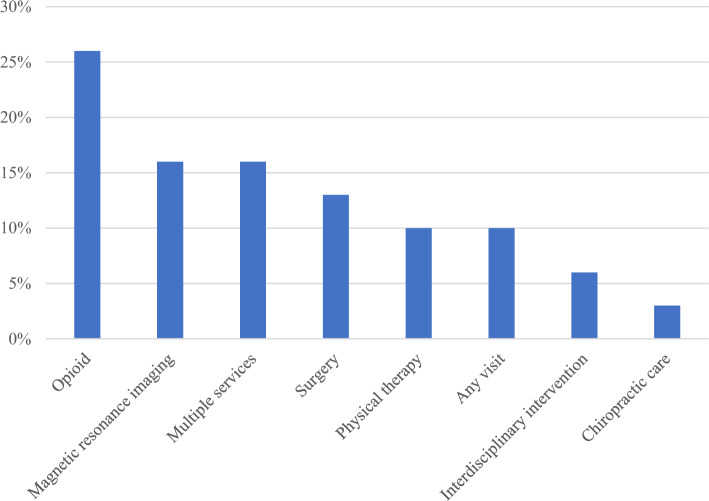
The type of services included (*n*=31)

Services included in the eligible studies were: opioids in (*n* = 8) studies [50, 55, 59, 61, 62, 72, 73, 75], magnetic resonance imaging (MRI) in (*n* = 5) studies [51, 52, 58, 60, 64], multiple (combination of services) in (i = 5) studies [49, 56, 69–71], surgery in (*n* = 4) studies [40, 41, 63, 65], physical therapy care in (*n* = 3) studies [66, 68, 74], visits to any healthcare provider in (*n* = 3) studies [47, 48, 54], interdisciplinary biopsychosocial intervention in (*n* = 2) studies [53, 67], and chiropractic care in (*n* = 1) study [57]; Fig. 3 .

### Factors Affecting Time to Service

Eight studies identified the factors associated with time to service [40, 49, 55, 69, 70, 72, 75, 77]. These included individual, workplace, injury, and health service-related factors and are described in the following section (Table 3).

**Table 3:** Factors affecting time to service (n = 8)

Factors			Service	Reference
Individual	Gender	Male	Opioid±	Carnide et al. 2020 [72]
			MRI±	Graves et al. 2012 [77]
			Multiple*‡	Blanchette et al. 2017 [70]
				Cote et al. 2005 [49]
		Female	Surgery†	Ren et al. 2020 [40]
	Age	Older age	Opioids±	Carnide et al. 2020 [72]
			Surgery†	Ren et al. 2020 [40]
			Multiple†	Blanchette et al. 2017 [70]
				Razmjou et al. 2015 [69]
	Income	Higher personal	Multiple†	Blanchette et al. 2017 [70]
	Remoteness	Being rural or urban/rural mixed	Opioid±	Carnide et al. 2020 [72]
	Functional limitation	Higher functional limitations (higher scores on the Roland-Morris scale	Multiple±	Cote et al. 2005 [49]
	Comorbidities	Having comorbidities	Opioid±	Carnide et al. 2020 [72]
	Pain	Pain radiated below the knee		Stover et al. 2006 [55]
	Tobacco use	Using tobacco compared to never tobacco user		
Work-related factors	Availability of return to work programme	Return to work programme available	Multiple‡	Blanchette et al. 2017 [70]
	Employers’ doubt about the work-relatedness of the injury	Employer’s doubt about the work-relatedness of the injury and		
	Number of employees	Greater number of employees in the workplace		
	Legal representation	Having had legal representation	Surgery†	Ren et al. 2020 [40]
	Occupation	Heavy physically demanding occupation	Opioid‡	Carnide et al. 2020 [72]
		Clerical/sales workers compared to service occupations workers		Stover et al. 2006 [55]
Injury-related factors	Injury severity	Greater injury severity	Opioid±	Gross et al. 2009 [75]
				Carnide et al. 2020 [72]
				Stover et al. 2006 [55]
			MRI±	Graves et al. 2012 [77]
			Multiple‡	Cote et al. 2005 [49]
				Blanchette et al. 2017 [70]
	Previous compensation history	Previous history of compensation claim		
	Year of injury	More recent year of injury	Opioid†	Carnide et al. 2020 [72]
Healthservice-related factors	Type of provider	Initial provider was pain related	Opioid±	Carnide et al. 2020 [72]
		Surgeon as an initial contact compared with a primary care	MRI‡	Graves et al. 2012 [77]
		An initial provider was Chiropractor		
		First line provider was physiotherapist	Multiple†	Blanchette et al. 2017 [70]
	Prescriber demographics	Female first line provider	Opioid†	Carnide et al. 2020 [72]
		Visiting young prescribers		

*= more than one service involved, ± time to service is shorter for the factor, † time to service is longer for the factor, ‡ time to service is mixed, n Number of studies, MRI magnetic resonance imaging

#### Individual-Related Factors

##### Gender

Five studies found a significant relationship between gender and the time to service [40, 49, 70, 72, 77]. Of these, three studies indicated male workers were associated with shorter time to service. The studies involved multiple services (i.e. medical doctor, chiropractor, physiotherapist, and nurse practitioner) used within a month, MRI within six weeks, and early opioid use within eight weeks of low back pain onset. A single study involving multiple services for low back injuries showed that males were more likely to receive delayed services [49], while another single study indicated that female workers received delayed surgery for back pain [40].

##### Age

Four studies found a significant relationship between age and time to service [69, 70, 72, 79]. Three studies found that older age was associated with longer time to service [40, 69, 70]. These three studies involved multiple services (i.e. medical doctor, chiropractor, physiotherapist, and nurse practitioner) and, surgery for low back pain, and assessment and clinical investigations for a shoulder injury as the service types. The fourth study reported that older workers used opioids early (i.e. within eight weeks) of low back injury [72].

##### Personal Income

A single study found that the time to service (i.e. first-line service involving a medical doctor, chiropractor, nurse practitioner, and physical therapist) for low back pain was more likely longer in high-income workers than in low-income workers [70].

##### Remoteness

One study of low back pain in workers found an association between residence in rural or urban/rural mixed regions and a shorter time to opioid use (i.e. within eight weeks of injury) [72].

##### Comorbidity

A single study demonstrated that workers with comorbidities used opioids soon (i.e. within eight weeks of a back injury) [72].

##### Tobacco Use

A single study found that workers with low back pain who used tobacco daily received opioids earlier (i.e. within six weeks of their initial healthcare visit) than workers who never used tobacco [55].

##### Functional Limitation

A single study found that experiencing a higher functional limitation was associated with a shorter time to service for low back pain (i.e. within the first four to sixteen weeks of injury) [49].

#### Work-Related Factors

##### Return to Work Programme

A single study found that the availability of a return-to-work programme in the workplaces was associated with a shorter time to services (first-line service) than workplaces with no return-to-work programme available [70].

##### Occupation

A single study reported that patients with low back pain whose occupation was clerical/sales experience a longer time to service (any visit to a provider) [49].

##### Employers' Doubt About Work-Relatedness of Injury

In one study, it has been observed that time to service was longer among workers with low back pain whose employers had doubts about occupational relatedness of injury [70].

##### The Number of Employees

One study showed that workers with low back pain in workplaces with a high number of employees experience a longer time to provider visits [70].

#### Injury-Related Factors

##### Injury Severity

Five studies demonstrated that injury severity was significantly associated with time to service [49, 55, 70, 72, 75, 77]. Three of the five studies indicated that time-to-service was shorter among the workers with greater injury severity. Of these studies, two studies involved early opioid use for low back pain [55] and for fractures, dislocations, and amputations [75], and another study involved early MRI for radiculopathy [77]. In contrast, two studies found that greater severe injuries were related to delayed services for low back conditions (visit to provider in both studies) [49, 70].

##### Previous Compensation History

A single study found that workers with a prior claim history had a shorter time to service for low back pain [70].

##### Pain

In one study, workers with greater pain severity were associated with early opioid use (i.e. within six weeks of healthcare visit) for low back conditions [55].

##### Year of Injury

A single included study involving early opioid prescription found that a more recent year of injury was associated with a longer time to service [72]. Two other included studies also reported decreasing trends in early opioid use with an increasing year of injury, but only descriptive results were documented [73, 75].

#### Health Service-Related Factors

##### The Type of Provider

One study showed that having a physiotherapist as the initial provider was associated with a longer time to service [70]. In another study, first consulting a surgeon was associated with early MRI utilization for low back pain (i.e. within six weeks of injury) [77].

##### Prescriber Demographics

Time to opioid use was shorter (i.e. within eight weeks of a low back injury) for workers who had their first visit with female and younger prescribers in a single study [72].

Overall, most studies examining the factors influencing the timing of services used quantitative administrative datasets and primarily focused on the characteristics of the injured workers. There was no qualitative study reported, and the factors related to health systems (e.g. the availability of providers), compensation schemes, insurance policies, and employers were insufficiently addressed.

## Time to Service as a Predictor of Worker Outcomes

Table 4 provides a summary of outcome measures and definitions. There was a significant association between time to service and worker outcomes in (*n* = 25) studies [40, 41, 47, 48, 50, 52–54, 58–64, 66–71, 73–75, 77]. The majority of eligible studies that assessed the association between time to service and worker outcomes were retrospective designs, that involved low back conditions, and most studies were limited to North America. Studies on the relationship between time to service and patient-reported health outcomes, including mental health (e.g. depression and anxiety) were limited [65], and no studies were identified regarding addiction as an outcome among early opioid users. The timing definitions and outcome measures were also used inconsistently across studies.

**Table 4:** Summary of the association between time to service and outcomes (n*=27)

Author (year) and Region	Design and sample size (n)	Conditions	Service and timing	Outcome	Outcomes definitions	Relationship with timing
Besen et al. 2016 [47] (USA)	Retrospective cohort; n = 64,004	Low back pain	0 days medical service lag (reference group), 1 to 3 days lag, 4 to 6 days lag, 1 week up to 2 weeks lag, 2 weeks up to 30 days lag, 30 days up to 60 days lag, and 60 days up to 1-year lag (any visit)	Length of disability	The length of disability was calculated from the date that a claimant first took temporary partial disability (TPD) or temporary total disability (TTD) until the date at which TPD or TTD ended. TPD or TTD was considered to have ended when no disability days were taken for at least 14 days consecutively. For claims in which the length of disability exceeded 1 year, the value was top-coded at 365 days	The shorter time interval between injury and health use is associated with lower disability duration
Besen et al. 2018 [48] (USA)	Retrospective cohort; n = 76,067	WRMSD and Fractures injured	Service (any visit); Over 85% of the fracture claimants received medical care within a day of injury, whereas less than half of the WRMSD claimants did (41%).	Length of disability	The number of days from the date that a claimant first took paid lost work time until the date at which paid lost work time ended	A shorter time interval between injury and first service was associated with a shorter length of disability
Blanchette et al. 2017 [70](Canada)	Retrospective cohort; n = 5520	Back pain	Longer to initiate service (first-line services including medical doctors, physiotherapist, Chiropractor, and nurse practitioner)	Compensation duration	The duration of the first episode of full compensation after the first healthcare consultation	Delayed the end of compensation duration
Busse et al. 2015 [71] (Canada)	Observational cohort study; n = 1442	Acute low back	Opioid received early within four weeks of claim approval, compared to early physiotherapy and chiropractic care received within four weeks	Claim duration /time to claim closure	The duration in days from disability claim approval until the claim was closed	Associated with longer claim duration [Hazard Ratio = 0.68; 99% CI (0.53 to 0.88)]
Carnide et al. 2019 [73] (Canada)	Historical cohort; n = 55,571	Acute and subacute nonspecific low back pain disorder	Opioid received early within eight weeks after injury date	Work disability duration	The total number of days receiving short-term disability benefits from WorkSafe British Colombia after the 8-week exposure window and up to 52 weeks after injury. Receipt of at least 1 day of short-term disability benefits (yes/no) in this same outcome window was also constructed	Prolonged work disability associated with opioid compared with nonsteroidal Anti-Inflammatory Drugs and Skeletal Muscle Relaxants use
Ehrmann-Feldman et al.1996 [74] (Canada)	Retrospective cohort; n = 2147	Acute back injury	Physical therapy service received early within the first 30 days following the injury onset	Return to work	Return to work was dichotomized as absence from work for less than 60 days represented an early return to work, whereas absence from work of more than 60 days represented a late return to work	Had a strong protective effect on return to work within 60 days.
Faour et al. 2017 [41] (USA)	Retrospective cohort; n = 1509	Neck (Degenerative Disc Disease and Radiculopathy)	Surgery (Multi level Cervical Fusion) for Radiculopathy neck Pain with Radicular Symptoms within > 2 years of the injury date	Return to work	Patients were considered to have successfully returned to work (stable RTW), if they returned to work and maintained continuous ‘‘at-work’’ status for at least 6 months within a 3-year period after surgery	injury-to-surgery > 2 years was negative a predictor of stable return to work status for patients who underwent multilevel cervical fusion
Franklin et al. 2008 [50] (USA)	Prospective cohort; n = 1843	Back injury	Opioids received early, within the first six weeks of the injury date	Work disability duration	Work disability was the receipt of wage replacement benefits for temporary total disability 1 year (365 days) after claim receipt	Associated with long-term disability
Graves et al. 2014 [52] (USA)	Prospective cohort; n = 1770	Acute and sub-acute low back pain	MRI received early (nonadherent) within six weeks of accident date	Cost Healthcare utilization	The authors used the Current Procedural Terminology (CPT-4) codes and codes specific to L&I (“local codes”) to determine healthcare utilization and costs incurred for 1 year following the injury date	Lumbosacral injections or surgery utilization increased costs (e.g. outpatient, inpatient, and nonmedical services, and disability compensation) all increased.
Graves et al. 2012 [77] (USA)	Prospective cohort; n = 1830	Acute back sprain/strain	Early MRI received within 42 or less days/ within six weeks after injury date	Injection	The study calculated the proportion of workers who received a spinal injection in 30 days after the early MRI	Lead to the subsequent injection
Greenwood et al.1990 [53] (USA)	Controlled design n = 284 (121 = experimental group and 163 control group)	Acute back injury	Case management delivered very early within two weeks after the injury date	Length of disability in days Disability cost Medical cost	Definition not available	Increased medical cost The length of disability and disability cost did not decrease
Gross et al. 2009 [75] (Canada)	Historical cohort study; n = 137,175	Back and other sprain/strain, fracture, amputation, burn, or dislocation; acute compensated workers	Early opioid received within first two weeks of claim acceptance	Recovery	The study used recovery as a surrogate indicator of sustained return to work after injury, cumulative days receiving wage replacement benefits measured up to 1 year after the initial injury date, and included all total temporary disability payments	Associated with delayed recovery
Haight et al. 2020 [62] (USA)	Retrospective cohort; n = 83,150	Musculoskeletal conditions including lower extremity, upper extremity, back/neck, multiple part)	Early opioid received within six weeks of injury date	Work disability duration	Binary measures of work disability outcome were employed: (1) receiving more than 90 days of time loss following the date of injury; (2) receiving more than 1 year of time loss; (3) receipt of a Department of Labor and Industries pension for total permanent disability (workers’ compensation pension); and (4) a measure for receipt of income support from Social Security Administration, recorded by Department of Labor and Industries in a Social Security offset data field	Significantly and substantially associated with a long-term temporary and permanent disability
Heins et al. 2016 [61] (USA)	Retrospective cohort; n = 123,096	Back and Shoulder	Early opioid prescription dispensed within a month of the injury date	Long-term opioids use	An average of at least one prescription per month for three months or at least three consecutive prescription refills with less than 1 month between refills	Associated with significantly lower risk of long-term opioid use in back injury Associated with a greater risk of long-term opioid use in shoulder injury
Lavin et al. 2013 [63](USA) (USA)	Retrospective cohort; n = 582	Low back pain (lumbar spinal)	Surgery received early, within less than a year of the injury date	Claim costs	Cost determined according to the injury severity and outcomes and categorized as: - (1) minor claims (initial reserve less than $15,000 and final cost less than $100,000); (2) migrated catastrophic claims (initial reserve less than $15,000 and final cost $100,000 or greater); (3) false catastrophic claims (initial reserve of 15,000 or greater and final cost less than $100,000); and (4) true catastrophic claims (initial reserve $15,000 or greater and final cost $100,000 or greater)	Decreased the outcomes
				Lost time work	Measured using the wage loss method of calculating indemnity payments (Temporary/total benefits are paid until the injured employee returns to work, i.e. claims duration = lost time days)	
Mahmud et al. 2000 [64] (USA)	Retrospective cohort; n = 98	Low back pain	Early imaging (within 30 days of injury)	Disability duration.	The number of compensated days lost from work/is calculated by dividing the total amount of indemnity payment by the average weekly rate (a percentage of the average weekly wage established by each state)	Associated with increased disability duration.
Patel et al. 2022 [65] (USA)	Retrospective cohort; n = 193	Degenerative spinal disease	Minimally invasive transforaminal lumbar interbody fusion; service received within < 90 days, 90–179 days, and > 180 days) from the first day of the appointment for Surgery.	Pain Disability Mental health Physical Function	The study cited previous evidence to indicate the measurements tools used [102, 103]	No effect on outcomes
Phillips et al. 2017 [66] (USA)	Prospective pilot study; n = 75	Upper extremity, lower extremity, neck, back, and other	Early physical therapy received at the point of initial injury care	Duration of care	The elapsed time between the patient’s first visit to the Occupational Medicine department and the last day of care	Decreased duration of care and claim costs
				Claim costs	Workers’ compensation claims at least $5000 were reviewed for trends related to potential sources of delay in care and increased incurred costs	
Razmjou et al. 2015 [69] (Canada)	Retrospective cohort; n = 550	Shoulder injury	Early multidisciplinary service received within 16 weeks of injury onset	Pain improvement	The Numeric Pain Scale (NPS) was used to assess pain status. The NPS uses a 0 to 10 scale, with 0 being no pain and 10 being the worst imaginable pain	Associated with less exacerbated pain behaviours
Ren et al. 2020 [40] (USA)	Retrospective cohort; n = 791	Chronic LBP (spondylolisthesis)	Surgery within two years of injury date and after two years.	Return to work	Returning to work within 2 years after Surgery and remaining working for at least 6 months	Surgery within 2 years was more likely to return to work have fewer days absent from work lower medical costs, and fewer sessions of psychotherapy, physical therapy, and chiropractic care.
				Days absent from work	The mean of days lost work	
				Medical costs	The medical cost was calculated as a mean of costs for claims with Surgery ≤ 2 and > 2 years and compared	
				Healthcare utilization	Healthcare utilization was the rate of healthcare utilized following Surgery	
Schultz et al. 2013 [67] (Canada)	Randomized controlled trial (RCT); n = 63	acute and subacute low back pain injury	Early multimodal intervention within four to ten weeks of injury onset compared to usual case management (biopsychosocial approach)	Return to work	Return to work was a binary outcome measured at 3, 6, and 12 months	Greater indication of a return to work success
Sinclair et al.1997 [68] (Canada)	Prospective cohort; n = 885	Soft tissue acute musculoskeletal conditions including the back, upper or lower limb	Early physical therapy (treatment is a treatment used within two days after the injury as early as possible but can also be as late as up to seventy days of injury) compared to usual care	Duration of benefits	Duration of benefits was the cumulative number of calendar days receiving 100% benefits within 365 days after an accident	No effect on the duration of benefits The healthcare cost higher Functional status, health-related quality of life, and pain measures all improved
				Healthcare cost	Healthcare cost was extracted from the Workers’ Compensation Board files	
				Functional status	Functional status was measured by the Roland Morris questionnaire (scale ranging from 0, no disability, to 23, indicating severe disability) [104]	
				Health-related quality of life	Health-related quality of life was measured by the generic health-related quality of life measure Short Form (SF-36) with eight domain scores. Each domain scored from 0 (poor health) to 100 (good health).	
				Pain	Pain measures were assessed by the Von Korff pain measures. The pain grade score was obtained by averaging the response to the first three of Von Korff’s questions and rescaling the results so that 0 represents the lowest pain and 100 represents the highest pain [105]	
Sinnott et al. 2009 [54] (USA)	Retrospective cohort; n = 35,304	Acute, nonspecific low back pain	Service (any visit) received within ≤ 14 days; 14 and ≤ 28 days; 28 and ≤ 56 days; > 56 and ≤ 84;> 84 and ≤ 182 days; and > 82 days (service not stated) .	Chronic disability (receiving 91 days or more of temporary disability benefits)	Receiving 91 days or more of temporary disability (TD) benefits The number of TD days paid for each claim was calculated by dividing the total dollars paid to the injured worker in TD benefits by the average weekly wage of that injured worker, restricted by the State-regulated allowable minimum and maximum benefits specific to the month and year the worker was injured	Delaying medical treatment from 2 to 4 weeks resulted in 50% higher odds of chronic disability [AOR: 6.467, 95% CI (5.609–7.455)].
Wasiak et al. 2007 [57] (USA)	Retrospective cohort; n = 6019	Nonspecific, uncomplicated low back pain	Chiropractic received within 30 days of onset	Duration of work disability	The authors used compensation payments for lost work time measurement	The association is independent of each other
				Recurrence of work disability	Resumption of payments for total work disability after a minimum of a 3-day break in compensation payments, implying at least a temporary return to work	
Webster et al. 2007 (USA) [59]	Retrospective cohort; n = 8443	Low back pain	Opioid received early, within 15 days post-onset	Disability duration	The average weekly payments (wage replacement) truncated at 730 days	Increased all outcomes
				Medical costs	The paid-to-date total for closed claims (90.2% of the claims) and the estimated total medical costs that include paid-to-date plus reserved costs for open claims	
				Late opioids	Five or more opioids prescriptions between 30- and 730-day post-onset	
				Subsequent Surgery	surgical procedures within two years post-onset identified from the Physician’s Current Procedural Terminology (CPT) billing codes database	
Webster et al. 2014 [60] (USA)	Retrospective cohort; n = 3022	Acute back pain	Early MRI received within 30 days of the injury date	Healthcare utilization	Healthcare utilization post-MRI was identified using Clinical Procedural Terminology codes and was evaluated at 3, 6, 9, and 12 months post-MRI. Subsequent healthcare utilization included advanced imaging, injection, and Surgery Total medical costs, based on paid medical bills, were computed for each subgroup	Significantly associated with a large and sustained escalation in medical costs, reflecting increases in overall medical utilization.
				Medical costs	Costs for the pre-MRI periods were aggregated from the date of onset to the first 14 days for the no MRI groups, up to 30 days post-onset for the early MRI groups, and between 42 and 180 days for the timely MRI groups. Post-MRI costs were aggregated from the day after MRI (or the 16th day for the no MRI groups) to the 3-, 6-, 9-, and 12-month follow-up dates. All totals exclude MRI costs	
Webster et al. 2010 [58] (USA)	Retrospective cohort; n = 3264	Disabling acute low back pain	Early MRI received within 30 days of injury	Disability duration	The post-MRI disability duration was calculated as the number of consecutive days of paid indemnity after the MRI to the last continuous indemnity payment date. Disability duration post-MRI for the no-MRI group was calculated from the claim onset to the end of the first disability episode. All disability durations were truncated at the end of the 2-year study period	Associated with worse disability and increased medical costs and Surgery
				Medical costs	Medical costs post-MRI were calculated from the time post-MRI to the end of the 2-year study period. These costs were based on the paid-to-date medical services total and truncated at the end of the 2-year study period. The medical costs post-MRI for the no-MRI group were calculated from 14 days post-onset to the end of the study period	
				Surgery	Receipt of Surgery Post-MRI Cases who underwent Surgery within 2 years post-onset were identified by Current Procedural Terminology (CPT) codes related to lumbar surgical procedures	

AOR = Adjusted Odds Ratio CI: Confidence Intervals IRR = Incident Rate Ratio LBP = Low back pain MRI = Magnetic Resonance Imaging n^*^=Number of studies n = Sample Size NSAD = Non-Steroidal Anti-Inflammatory Drugs SMRS = Skeletal Muscle Relaxants TD: Temporary Disability TPD = Temporary Partial Disability TTD = Temporary Total Disability USA = United States of America WRMSD = Work-Related Musculoskeletal Disorder

In a study involving early physical therapy (i.e. service initiated at the initial point of healthcare contact) for upper and lower extremities, neck, back, and other body parts, time to service was associated with reduced costs and a shorter duration of care [66]. Another study involving early physical therapy received within 30 days [74] and one further study involving early evidence-based case-managed interdisciplinary (biopsychosocial approach) received within four to ten weeks of low back pain injury was associated with a greater rate of return to work [67]. Moreover, early physician assessment within four to sixteen weeks for shoulder injury was associated with improved patient-reported health outcomes, such as reduced pain exacerbation) [69].

Studies involving early opioid use (seven studies with various conditions) [50, 59, 61, 62, 71, 73, 75] and MRI (five studies with low back conditions) [52, 58, 60, 64, 77] demonstrated associations with longer duration of disability, increased costs, higher healthcare utilization, and poor patient-reported health outcomes. Moreover, a delayed visit to any provider for low back condition [54, 70] and surgery for neck injury (i.e. injury-to-surgery > 2 years) [41] was associated with negative outcomes (i.e. increased disability duration for a back condition and a decreased rate of return to work for neck injury).

Most studies reported more than one outcome. Therefore, we grouped the outcomes into related themes: work, cost, healthcare utilization, and patient-reported health outcomes. Each theme is discussed below.

### Work Outcomes

The work outcome measures include work disability duration and return to work.

#### Work Disability Duration

The relationship between time to service and work disability duration was reported in sixteen studies [40, 47, 48, 50, 54, 57–59, 62–64, 68, 71, 73, 75, 76]. Studies described work disability duration using time to claim closure, length of disability, compensation duration, claim duration, duration of benefits, days absent from work, days lost work, and lost time work. Some studies used the concept of indemnity/wage replacement benefits to measure work disability duration [57, 59, 63, 64, 68, 71, 73, 70].

Studies involving early opioid use for low back conditions [50, 59, 71, 73] and for the lower extremity, upper extremity, back/neck, or multiple body parts [62], and early MRI for low back pain [58, 64] indicated a prolonged disability duration.

Five included studies found that early timing of health services is associated with reduced disability duration [40, 47, 48, 63, 67]. One study found that early interdisciplinary intervention (i.e. 4–10 weeks of low back pain onset) was associated with a decreased average number of days lost from work [67]. A detailed report is presented in Table 4.

#### Return to Work

The relationship between time to service and a return to work (RTW) outcome was reported in four studies [40, 41, 67, 74]. Two studies of participants with low back pain found a faster RTW outcome for time to surgery within two years of injury, compared to surgery performed two years after the injury [40, 41]. A cohort study of physical therapy in Canada (Quebec) revealed that physical therapy received early (i.e. within 30 days of low back pain) resulted in shorter time to RTW than those who did not receive physical therapy early [74]. Another randomized controlled trial report in Canada [67] also found that workers who received early (i.e. 4–10 weeks of a back injury) a biopsychosocial model-based interdisciplinary intervention exhibited significantly better RTW outcomes.

### Claim Costs Outcomes

Nine studies reported the association between time to service and claim costs. The studies involving early opioids (i.e. 15 days within injury date) [59] and MRI (i.e. 6 weeks within injury report ) [52, 58, 60] for low back pain, and early physical therapy (i.e. within two days as soon as possible or as late as 70 days of injury ) for soft tissue acute musculoskeletal conditions in the back, upper, or lower limbs [68], and early multidisciplinary services for low back pain [53] were associated with increased medical and nonmedical costs.

Two studies indicated that surgery for low back pain within the first two years compared to surgery after two years [40, 63], and early physical therapy (i.e. physical therapy received at the initial point of care after injury report) for multiple body regions, was associated with decreased costs [66].

The effect of timing on cost outcomes was reported inconsistently. The majority of the studies reviewed addressed the relationship between early services and low back pain.

### Healthcare Utilization Outcomes

Eight eligible studies assessed the relationship between time to service and overall healthcare utilization outcomes. Healthcare utilization outcomes reported in eligible studies include the duration of care, late opioid use (five and above opioids prescriptions between 30- and 730-day post-onset, subsequent surgery, spinal injection (i.e. caudal, facet lumbar/sacral, transforaminal lumbar/sacral, or sacroiliac joint injections), and overall healthcare utilization (e.g. frequency of visit, and intensity).

In one study, a decreased risk of long-term opioid use (i.e. an average of at least one prescription per month for three months or at least three consecutive prescription refills with less than one month between refills) has been shown among workers with low back pain using opioids early (i.e. within one month of injury date) and an increased risk among workers with shoulder injuries [61]. In another report, early opioid use (i.e. within 15 days of injury) for low back pain was associated with increased rates of subsequent opioid use and surgery services [59]. Four included studies indicated that early MRI (i.e. within 30–42 days of injury) for acute low back pain resulted in increased likelihood of spinal/ Lumbosacral injection and overall health care utilization [52, 58, 60, 77].

Generally, the studies indicated that early utilization of opioids and MRI were associated with the increased likelihood of greater healthcare utilization.

### Patient-Reported Health Outcomes

Four included studies reported the association between time to service and patient-reported health outcomes [65, 68, 69, 75]. Patient-reported health outcomes included recovery, pain, health-related quality of life, mental health, and functional status. One eligible study involving surgery for low back pain (degenerative spinal disease) reported no significant relationship between time to service and pain, disability, mental health, and physical function [65]. Another single report found that early physical therapy (i.e. within as soon as two days or as late as seventy days of injury) for soft tissue musculoskeletal conditions, including the back, upper and lower limbs, was associated with improved pain, quality of life, and functional status [68]. Further, one included study showed that early opioids (i.e. within two weeks of claim acceptance) for back and related conditions were associated with delayed recovery [75]. Another study reported that time to early physician assessment (i.e. within 16 weeks of injury) for shoulder injury was associated with reduced pain symptoms [69].

## Discussion

This scoping review identified a wide range of individual, injury, workplace, and health service-related factors associated with time to service in eight included studies. The relationship between time to service and worker outcomes was observed in twenty-five studies, and four categories of outcomes were identified across the studies: work outcomes (i.e. disability duration and return to work), healthcare utilization, claim costs, and patient-reported health outcomes. A shorter time to physical therapy care and interdisciplinary biopsychosocial interventions after injury report were associated with positive worker outcomes such as reduced pain, shorter time to return to work, lower likelihood of subsequent healthcare use, improved functional capacity, and decreased healthcare and indemnity cost. Conversely, early opioids use and MRI after injury reporting, against guideline recommendations, resulted in a longer duration of disability, increased costs, and healthcare utilization, and poorer patient-reported health outcomes in workers’ compensation accepted claims for musculoskeletal conditions.

### Description of Time to Services

We noted variability in measuring and defining time to services. This variation may be because our review included any study that examined the timing of services. Moreover, the differences could be because the included studies used different milestones as starting points for the services, such as injury date, claim acceptance date, or initial point of care. Further, the included studies also employed different units of measurement for time to services, such as days, weeks, or months. A previous systematic review study by Arnold et al. supports the heterogeneity observed in our findings [34]. Arnold et al. measured the timing of physical therapy for acute low back pain and found varying definitions of early and delayed timing in the documented evidence. Authors of the study defined early as within 30 days of index date compared to delayed or usual care. Another prior systematic review study by Ojha et al. [25] found that most studies they examined defined early physical therapy as a service initiated within 14 days of the injury or index visit for musculoskeletal conditions.

We also found numerous studies that reported services as being accessed ‘early’ compared to not early. A possible explanation may be that studies that included services such as MRI and opioids typically involved early as defined by certain clinical practice guidelines. In our scoping review, 74% of the included studies examined low back pain conditions- a common musculoskeletal condition [80], most of which measured the prevalence and impact of the non-guideline adherent timing of certain services, including MRI and Opioids. Low back pain represents a substantial portion of workers’ compensation claims, so studies testing whether healthcare is guideline adherent are not necessarily surprising. These studies identified that services were frequently offered during the acute phase of low back conditions, even when such services were not in line with practice guideline recommendations. Furthermore, many studies reporting the timing of services relative to best practice care/ guidelines involved workers with low back pain [53, 67, 74].

### Factors Influencing Time to Services

Addressing the factors associated with health service timing could help identify the barriers to timely access to appropriate services. In our review, time to service was shorter for male workers experiencing low back pain in studies involving services with visits to any provider, early MRI, and opioids [70, 72, 77]. This report was in line with the findings of prior research [81, 82]. It has been shown that male workers are more likely to experience injuries and fatalities than females [83], which may be a possible reason for male workers to seek healthcare services earlier than females. Our study also found that the time to service was longer for female workers in a study involving surgery for low back conditions [40]. It has previously been reported that females experience a longer time to health services due to facing more barriers, such as family responsibility [84]. Another study also supports the finding of our review in that women workers experienced a longer waiting time for consultation and surgical treatment for a compensable shoulder injury, suggesting that the difference may be due to the combination of biological and social differences [85]. The current scoping review also demonstrated that other non-modifiable factors including older age were associated with time to service [40, 69, 70, 72]. Moreover, the time to opioid use was shorter for low back pain patients with comorbidities and those in rural and remote areas [72]. People with comorbidities may experience greater pain symptoms, leading to prompt healthcare-seeking practice.

Workplace factors, such as the availability of early return to work programmes within organizations, were associated with a shorter time to service for low back pain [70]. This may be due to the fact that workers in workplaces with return to work programmes available may have better information and awareness on early injury reporting and health-seeking practices.

Included studies also indicated that the severity of injury significantly influences time to service [49, 51, 55, 70, 72, 75]. Earlier study shows that a greater injury severity is associated with a shorter delay in healthcare consultation [86]. In the current review, a more recent year of injury was significantly associated with a longer time to service in a study involving early opioid prescriptions [72]. The decreasing pattern of receiving early opioids may be attributed to the increasing awareness of the potential adverse effects of early opioids or temporal changes related to workers’ compensation policies regarding early opioid reimbursement. Of the studies screened, Gross et al. reported a decreasing trend of early opioid prescriptions, with rates declining from 6.7% in 2000 to 4.8% in 2005 [75]. Moreover, a study by Carnide and colleagues demonstrated a reduction rate of early opioid prescriptions from 20.3% in 1998/1999 to 13.2% in 2000/2009 [73]. However, the authors did not report the significance of the association with time to service. Of the health service-related factors, one study demonstrated that the time to service for low back pain was longer when the type of first provider is a physiotherapist [70]. There is a lack of studies to compare these findings.

The included studies rarely reported on factors associated with health systems (e.g. referral process, availability of services), and compensation policies (e.g. insurance coverage). It is unclear why these were not studied. It may be that most studies used administrative claims data or that data was taken from a single jurisdiction, meaning comparisons of health systems or insurance policies were not possible.

### Association Between Time to Service and Worker Outcomes

Early access to evidence-based services, such as physical therapy and case-managed interdisciplinary biopsychosocial interventions, was associated with improved outcomes (i.e. a shorter duration of disability/a higher rate of return to work, lower claim costs, decreased healthcare duration, and improved patient-reported health outcomes (e.g. improved functional status, quality of life, and less pain symptoms)). For example, a study by Ehrmann-Feldman et al. shows that physical therapy within a month (early) of a back injury that involves various treatments (i.e. exercise, heat, ultrasound, back education, manipulation, and transcutaneous electrical nerve stimulation) is associated with a higher rate of return to work within 60 days than physical therapy utilized later [74]. This positive association was consistent across conditions affecting various body parts, including the upper and lower extremities, neck, back, shoulder, and other parts. Consistent with the present study, previous studies demonstrated that early treatment with best practice services was associated with desired outcomes [25, 87–89].

In the present review, a prospective study by Sinclair et al. indicated that early physical therapy utilization was associated with increased claim costs [20]. This report contradicts with a study by Young et al. [90]. The differences could be related to differences in the definition of timing and study population. While timely, appropriate care has been promoted as a good thing for recovery in people with various musculoskeletal conditions, our review has highlighted the challenges with testing that theory. For example, the study by Sinclair et al. defined early physical therapy as active, exercise, and education programme based-intervention initiated within two days or as delayed as 70 days of soft tissue musculoskeletal injury onset compared to usual care [68], whereas the study by Young et al. defined early physical therapy as treatment started on the same day of provider contact or within 30 days of pain diagnosis [90]. Understanding the context and characteristics of patients who benefit the most from early physical therapy assists in clarifying these disparities and guides informed decisions.

The association between early services and outcomes varied depending on the type of services provided. For example, surgery within one year compared to surgery after one year [63] and surgery within two years compared to surgery after two years of low back injury [40] resulted in a faster return to work rate, shorter disability duration, lower claim costs, and reduced healthcare utilization. For a neck injury, a lower rate of return to work was observed for surgery two years after the injury than surgery within two years [41].

Studies involving early MRI and opioid prescription demonstrated negative worker outcomes. Prior research suggests that early MRI has been associated with unfavourable outcome [51, 91]. Guidelines discourage early timing of diagnostic imaging (e.g. MRI) for conditions such as low back pain in the absence of severe underlying conditions [92, 93]. Consistent with literature findings [27, 94], early opioid use was associated with worse outcomes, with consistent results across the studies. Early opioids may lead to opioid addiction and prolonged use [95]. We found no studies that reported the effects of early opioid use on addiction in workers with low back conditions. Future research may use a prospective study to investigate the relationship between early opioids and subsequent addiction in patients managed under workers’ compensation systems.

## Limitations of the Current Scoping Review

This scoping review included studies with an array of data sources. Administrative datasets were most common, likely due to our defined population. Some studies used a combination of datasets, including administrative data sets linked to medical records and population data, surveys, and patient interviews. Included studies also used different study designs, such as randomized controlled studies, prospective and retrospective designs with statistical adjustments that were used to control potential confounders and manage missing data. Moreover, the current scoping review covered a broad range of conditions and services, with a robust report on how the timing of healthcare services funded through the workers’ compensation system affects the outcomes of individuals suffering from musculoskeletal conditions.

Some limitations mentioned by the studies examined include limited reliability of administrative data and a lack of a direct measure of injury severity (e.g. pain, intensity, and functional limitations), or missed potential variables within workers’ compensation administrative data for controlling confounders [57, 70, 72, 75]. Besides, some studies were descriptive [56, 69], included small sample sizes [53, 56, 65, 67, 96], or were cross-sectional [49]. There were also inconsistent timing definitions and outcome measurements across the studies and services, which make it challenging to compare study reports. Consistent outcome measurements may enable cross-study comparison and findings synthesis. Furthermore, the relatively wide range of concepts covered in the current scoping review limits our ability to deeply explore each construct. Despite these limitations, the findings of the included studies demonstrated a significant relationship between various characteristics and time to services, and its effect on outcomes.

## Implications

Variability in outcome measures used by included studies highlights the need for standardization of measures of healthcare service timing. A consistent outcome measure could assist in comparing the timing of various services between healthcare systems and insurance systems for the management of musculoskeletal conditions, such as low back pain within the workers’ compensation system [97]. Moreover, several factors associated with the timing of health services for workers’ compensation system accepted claims of musculoskeletal conditions highlight the need to consider barriers to timely access to appropriate services, as well as characteristics that drive early services with little evidence support. The provision of interventions that consider the need for individual patient characteristics, such as age, gender, injury severity, occupation, and medical history (e.g. comorbidities), may enable to achieve favourable worker outcomes and reduce potential costs associated with workers compensation claims. Moreover, workers’ compensation policies need to ensure that strategies to reduce the practice of early services with negative effects are in place. This could be accomplished through awareness raising and educating providers and patients about the risks and benefits of early services that lack evidence of effectiveness, such as opioids and MRI, and providing adequate access to early services with superior outcomes, such as physical therapy. Graves et al. for example, found that implementing a utilization review programme for advanced imaging reduced the trends of services with minimal benefits, including MRI and injection [98]. The study also showed that the utilization review strategy was associated with substantially lower claim costs, shorter average durations of disability, and a lower percentage of workers on disability payments.

Subject to the common drawback of a scoping review, we did not assess the methodological quality of the included studies. Acknowledging the breadth of a scoping review, the wider nature of services and conditions contained in the review may affect the representativeness of the study to a particular group or service. Moreover, the study included only peer-reviewed journals published in English, which may lead to the study selection bias. Finally, because our search was limited to the workers’ compensation context, findings may not be translated to the general and uncompensated populations.

## Conclusion

This scoping review found that time to service for individuals with compensated musculoskeletal conditions was associated with several individual, injury, workplace, and health service-related factors. The majority of the studies indicated the relationship between time to service and worker outcomes, with early access to physical therapy and biopsychosocial interventions indicating an increased rate of return to work for low back conditions, reduced pain for shoulder injury, improved functional status, health-related quality of life, and pain symptoms for the back, upper or lower limb musculoskeletal conditions, decreased duration of care and claim costs in patients with upper extremity, lower extremity, neck, and back conditions. Conversely, early opioids and MRI use were found to be associated with prolonged disability duration, increased claim costs, poorer patient-reported outcomes, and a greater likelihood of subsequent healthcare use, with consistent reports across studies. This review suggests that there is a need to consider various individual and contextual factors and develop strategies to minimize the early use of opioids and MRI and promote access to early services with better outcomes (e.g. physical therapy and interdisciplinary biopsychosocial intervention) for the management of compensable musculoskeletal conditions. Further study may be required to explore the various contextual factors affecting health service timing and its impacts on compensation outcomes.

### Supplementary Information

Below is the link to the electronic supplementary material.

Supplementary material 1 (DOCX 17.2 kb)

## Abbreviations

CINAHL: Cumulative Index to Nursing and Allied Health Literature ICD:International Classification of Diseases
LBP: Low back pain
MRI: Magnetic resonance imaging
NSAIDs: Nonsteroidal anti-inflammatory drugs
PRISMA-ScR: Preferred Reporting Items for Systematic Reviews and Meta-analysis Extension for Scoping Reviews
RCT: Randomized controlled trial
USA: United States of America
WRMSD: Work-related musculoskeletal disorders
