# Supplementary material for: Time to Service and Its Relationship with Outcomes in Workers with Compensated Musculoskeletal Conditions: A Scoping Review
Source: J Occup Rehabil. 2024 Jan 12;34(3):522–54. doi: 10.1007/s10926-023-10160-0 (PMC11364620; doi:10.1007/s10926-023-10160-0)
Supplement: Supplementary file 1 — Supplementary material 1 (DOCX 17.2 kb) [file 10926_2023_10160_MOESM1_ESM.docx]

**Supplementary File 2**: Data charting table

| 1. **Study detail** | Report |
| --- | --- |
| First author /year: |  |
| Title: |  |
| Journal: |  |
| 1. **Study characteristics** |  |
| Setting: |  |
| Design: |  |
| Study sample: |  |
| Sample size: |  |
| Study inception period/year of injury: |  |
| 1. **Participant characteristics** |  |
| Inclusion: |  |
| Exclusion: |  |
| Follow-up: |  |
| Data collection/data source: |  |
| Age (mean+ Standard deviation): |  |
| Sex, n%: |  |
| Type of musculoskeletal conditions: |  |
| Symptom duration as reported by the author (ie. acute, sub-acute, or chronic): |  |
| 1. **Service characteristics** |  |
| Type of service: |  |
| Type of provider: |  |
| 1. **Nature of time-to-service** |  |
| Use of time to service (ie. independent, dependent, or both): |  |
| Type of timing measure (ie. continuous, categorical, or information based on certain guidelines such as “early” service): |  |
| Timing measure/unit (e.g. days, weeks, month, year): |  |
| Timing start point (e.g. the point from which timing was defined): |  |
| Duration of time to service (e.g. average for continuous timing measure or duration of the period): |  |
| 1. **Time to service as a dependent variable** |  |
| What factors were predictors where time to service is a dependent variable?: |  |
| 1. **Time to service as an independent variable** |  |
| Where time-to-service was a predictor, what outcome measures other than time-to-service were used?: |  |
| Outcome definition/measurement : |  |
| 1. **Study findings:** |  |
| 1. **Author conclusion :** |  |
| 1. **Reviewer Note:** |  |
